# ^68^Ga-PSMA 11 ligand PET imaging in patients with biochemical recurrence after radical prostatectomy – diagnostic performance and impact on therapeutic decision-making

**DOI:** 10.1007/s00259-017-3858-2

**Published:** 2017-10-26

**Authors:** B. Grubmüller, P. Baltzer, D. D’Andrea, S. Korn, A. R. Haug, M. Hacker, K. H. Grubmüller, G. M. Goldner, W. Wadsak, S. Pfaff, J. Babich, C. Seitz, H. Fajkovic, M. Susani, P. Mazal, G. Kramer, S. F. Shariat, Markus Hartenbach

**Affiliations:** 1 Department of Urology, Medical University of Vienna, Vienna, Austria; 2 Division of General and Pediatric Radiology, Department of Biomedical Imaging and Image-guided Therapy, Medical University of Vienna, Vienna, Austria; 3 Division of Nuclear Medicine, Department of Biomedical Imaging and Image-guided Therapy, Medical University of Vienna, Vienna, Austria; 4 Department of Urology and Andrology, University Hospital Krems, Karl Landsteiner University of Health Sciences, Krems, Austria; 5 Department of Radiation Oncology, Medical University of Vienna, Vienna, Austria; 6 Center for Biomarker Research in Medicine, CBmed GmbH, Graz, Austria; 7 Division of Radiopharmaceutical Sciences, Department of Radiology, Weill Medical College of Cornell University, New York, NY USA; 8 Clinical Institute of Pathology, Medical University of Vienna, Vienna, Austria; 9 Department of Urology, University of Texas Southwestern, Dallas, TX USA; 10 Department of Urology and Division of Medical Oncology, Weill Medical College of Cornell University, New York, NY USA

**Keywords:** Biochemical recurrence, Hybrid imaging, PET/CT, PET/MRI, Prostate cancer, PSMA ligand

## Abstract

**Objective:**

To evaluate the diagnostic performance of [^68^Ga]Ga-PSMA^HBED-CC^ conjugate 11 positron emission tomography (PSMA-PET) in the early detection of metastases in patients with biochemical recurrence (BCR) after radical prostatectomy (RP) for clinically non-metastatic prostate cancer, to compare it to CT/MRI alone and to assess its impact on further therapeutic decisions.

**Material and methods:**

We retrospectively assessed 117 consecutive hormone-naïve BCR patients who had ^68^Ga-PSMA 11 PET/CT (*n* = 46) or PET/MRI (*n* = 71) between May 2014 and January 2017. BCR was defined as two PSA rises above 0.2 ng/ml. Two dedicated uro-oncological imaging experts (radiology/nuclear medicine) reviewed separately all images. All results were presented in a blinded sequential fashion to a multidisciplinary tumorboard in order to assess the influence of PSMA-PET imaging on decision-making.

**Results:**

The median time from RP to BCR was 36 months (IQR 16–72). Overall, 69 (59%) patients received postoperative radiotherapy. Median PSA level at the time of imaging was 1.04 ng/ml (IQR 0.58–1.87). PSMA-positive lesions were detected in 100 (85.5%) patients. Detection rates were 65% for a PSA value of 0.2 to <0.5 ng/ml, 85.7% for 0.5 to <1, 85.7% for 1 to <2 and 100% for ≥2. PSMA-positive lesions could be confirmed by either histology (16%), PSA decrease in metastasis-directed radiotherapy (45%) or additional information in diffusion-weighted imaging when PET/MRI was performed (18%) in 79% of patients. PSMA-PET detected lesions in 67 patients (57.3%) who had no suspicious correlates according to the RECIST 1.1 criteria on MRI or CT. PSMA-PET changed therapeutic decisions in 74.6% of these 67 patients (*p* < 0.001), with 86% of them being considered for metastases-directed therapies.

**Conclusions:**

We confirm the high performance of PSMA-PET imaging for the detection of disease recurrence sites in patients with BCR after RP, even at relatively low PSA levels. Moreover, it adds significant information to standard CT/MRI, changing treatment strategies in a significant number of patients.

## Introduction

Prostate cancer (PC) is responsible for 11% of all cancer-related deaths [1]. Despite attempted local therapy with curative intent, up to 40% [2] of the patients experience disease recurrence. Most of these patients have prostate-specific antigen (PSA)-only recurrence without any clinical evidence of metastases [3]. Management of patients with PSA-only recurrence [i.e., biochemical recurrence (BCR)], is a clinical challenge as some may have localized disease only potentially benefiting from local therapy. Avoidance of overtreatment with its unnecessary side effects while ensuring effective durable disease control remains dependent on identification of the site of disease recurrence [4]. Indeed, early salvage treatment can confer durable local disease control, prolonging survival [5–7]. Patients with BCR after radical prostatectomy (RP) with curative intent are often offered salvage radiotherapy (RT) alone, androgen deprivation therapy (ADT) alone or a combination of these two [3]. Salvage RT is most effective when PSA levels are below 0.5 ng/ml [8–10]. At this threshold, the actual imaging techniques are limited in their sensitivity to differentiate local versus distant recurrence. Contrast-enhanced computerized tomography (CT) and bone scan (BS), the current standard diagnostic tests for staging of PC, have, unfortunately, inadequate sensitivity for the detection of PC in PSA ranges below 10 ng/ml [11].

The development of molecular imaging and the use of specific target probes, like choline-based positron emission tomography (PET), has already improved the diagnostic accuracy in patients with BCR after RP [12, 13]. Although the detection rates markedly increased when applying choline-based hybrid imaging [14, 15], the need for more accurate and earlier detection of metastases at lower PSA levels is necessary for the tailoring of individualized salvage therapies. In this context, [^68^Ga]Ga-PSMA^HBED-CC^ conjugate 11 (PSMA) has emerged as a promising radiopharmaceutical [16–18], albeit still not approved and, therefore, considered experimental [19]. Previous studies have shown that PSMA-PET imaging can detect lesions at lower median PSA levels. However, the verification of these lesions has not been assessed to our knowledge. Through verification, one can ensure that these lesions are not false positives and that all lesions have been detected. Moreover, we hypothesize that PSMA-PET demonstrates high sensitivities also at lower median PSA levels and contributes a major impact on the clinical decision-making process. Unfortunately, none of the previous reports, to our knowledge, addressed the clinical implications of this technology in the clinical decision-making regarding therapy. The aim of this study was to evaluate the diagnostic performance of PSMA-PET in the early detection of metastases in patients with BCR at lower PSA levels after RP for clinically non-metastatic PC and to assess its impact on therapeutic decision-making compared to standard imaging with CT/MRI.

## Material and methods

### Patients

145 consecutive patients, evaluated at our institution with PSMA-PET/CT (*n* = 68) or -PET/MRI (*n* = 77) for BCR between May 2014 and January 2017 were retrospectively analyzed. Initially, all patients were supposed to undergo PET/MRI. Nevertheless, patients with metal implants in the pelvic region were shifted to PET/CT due to unfavorable image quality and unknown effects on attenuation correction. Also, patients with implants not suitable for a 3-Tesla (T) system, claustrophobia and/or pain were shifted to PET/CT. A total of 28 patients were excluded due to initial treatment with RT of the prostate, leaving 117 patients for final analysis.

All patients were treated with RP according to guidelines recommendation. All surgical specimens were processed according to standard pathologic procedures, staged with the AJCC TNM classification and graded with the WHO/ISUP 2005 grading system [20].

BCR was defined as two consecutive PSA rises above 0.2 ng/ml. Follow-up was generally every 3 months for the first 2 years, then semiannually until the fifth year, then annually.

All reported investigations were conducted in accordance with the Helsinki Declaration and national regulations. The study was approved by the local Ethics Committee (permit 1440/17). [^68^Ga]Ga-PSMA^HBED-CC^ conjugate 11-PET was produced and injected according to a compassionate use during the conduct of a prospective clinical trial (EudraCT: 2014–004758-33; Clinicaltrials.gov Identifier: NCT02659527).

### Imaging protocol and analyses

PET/MRI was performed on a Biograph mMR (Siemens, Germany), capable of simultaneous data acquisition, consisting of a MRI-compatible PET detector integrated in a 3.0-T whole-body MRI scanner. The PET component uses a 3-dimensional (3D) acquisition technique and offers an axial field of view (FOV) of approximately 23 cm and a transversal FOV of 45 cm with a sensitivity of 13.2 counts per second/kBq.

Local PET of the pelvis comprised a 10-min listmode acquisition, starting 60 min after injection. Partial body PET (skull base to thigh) was performed with 4 bed positions, with a 4-min sinogram mode each. Reconstruction parameters for PET were: 3 iterations/21 subsets; summation of the 10-min pelvic acquisition for visual and semiquantitative analysis. MRI-based attenuation correction was applied using DIXON-VIBE sequences comprising in- and opposed-phase as well as fat- and water-saturated images.

The integrated 3-T MRI is performed with the following sequences and parameters: pelvis: T2w turbo spin echo (tse) axial: matrix size: 512 × 512, in-plane resolution: 1.1 × 0.8 x 5 mm; FOV: 263 × 350 mm; TR: 3600 ms; TE: 103 ms. T1w turbo spin echo (tse) coronal: matrix size: 384 × 384, in-plane resolution: 1.0 × 0.9 × 5 mm; FOV: 221 × 350 mm; TR: 600 ms; TE: 12 ms. Diffusion-weighted imaging (DWI): matrix size: 192 × 192, in-plane resolution: 2.6 × 2.0 × 5 mm; FOV: 285 × 380 mm; b-values: 0.600 s/mm^2^; TR: 9200 ms; TE: 85 ms. Partial-body MRI simultaneous with PET: T2w HASTE: matrix size: 256 × 256, in-plane resolution: 1.56 × 1.5 × 6 mm; FOV: 380 × 380 mm; TR: 1400 ms; TE: 121 ms. T1 VIBE matrix size: 195 × 320, in-plane resolution: 1.6 × 1.2 × 3 mm; FOV: 309 × 380 mm; TR: 4.56 ms; TE: 2.03 ms. Sagittal spine sequences after PET: T1 tse: matrix size: 320 × 320, in-plane resolution: 1.4 × 1.1 × 3 mm; FOV: 263 × 350 mm; TR: 666 ms; TE: 9.6 ms. T2 STIR: matrix size: 320 × 320, in-plane resolution: 1.4 × 1.1 × 3 mm; FOV: 263 × 350 mm; TR: 3500 ms; TE: 43 ms.

Sixty minutes before PET/MRI acquisition start, patients received an intravenous injection of of 2 MBq/kg body weight [^68^Ga]Ga-PSMA^HBED-CC^ conjugate 11 intravenously. For improved image quality, forced diuresis with 20 mg of furosemide was applied intravenously before the PSMA application and all patients in PET/MRI received a bladder catheter.

PET/CT was performed, from the vertex to the upper thigh, using a 64-row, multi-detector hybrid system (Biograph TruePoint 64; Siemens, Erlangen, Germany), with an axial FOV of 216 mm, a PET sensitivity of 7.6 cps/kBq and a transaxial PET resolution of 4–5 mm (full-width at half-maximum, FWHM). PET was performed 90 min after an intravenous administration of 2 MBq/kg body weight [^68^Ga]Ga-PSMA^HBED-CC^ conjugate 11 with 4 min/bed position, four iterations per 21 subsets, a 5-mm slice thickness, and a 168 × 168 matrix, using the point-spread-function (PSF)-based reconstruction algorithm TrueX. CT maps were used for PET attenuation correction. Venous-phase CE-CT was obtained after the intravenous injection of 100 ml of a tri-iodinated, non-ionic contrast medium at a rate of 2 ml/s; a tube voltage of 120 mA, a tube current of 230 kV, a collimation of 64 × 0.6 mm, a 3-mm slice thickness at a 2-mm increment, and a 512 × 512 matrix.

PET/CT and PET/MRI reviews were separately assessed by an experienced reader for prostate hybrid imaging (MH), and CT/MRI alone was also separately assessed by an uro-radiologist (PB). For MRI and CT alone, AGFA IMPAXX EE software was used; for PET/MRI and PET/CT, Hermes Hybrid 3D (Hermes Medical Solutions Stockholm) was used.

**Fig. 1:**
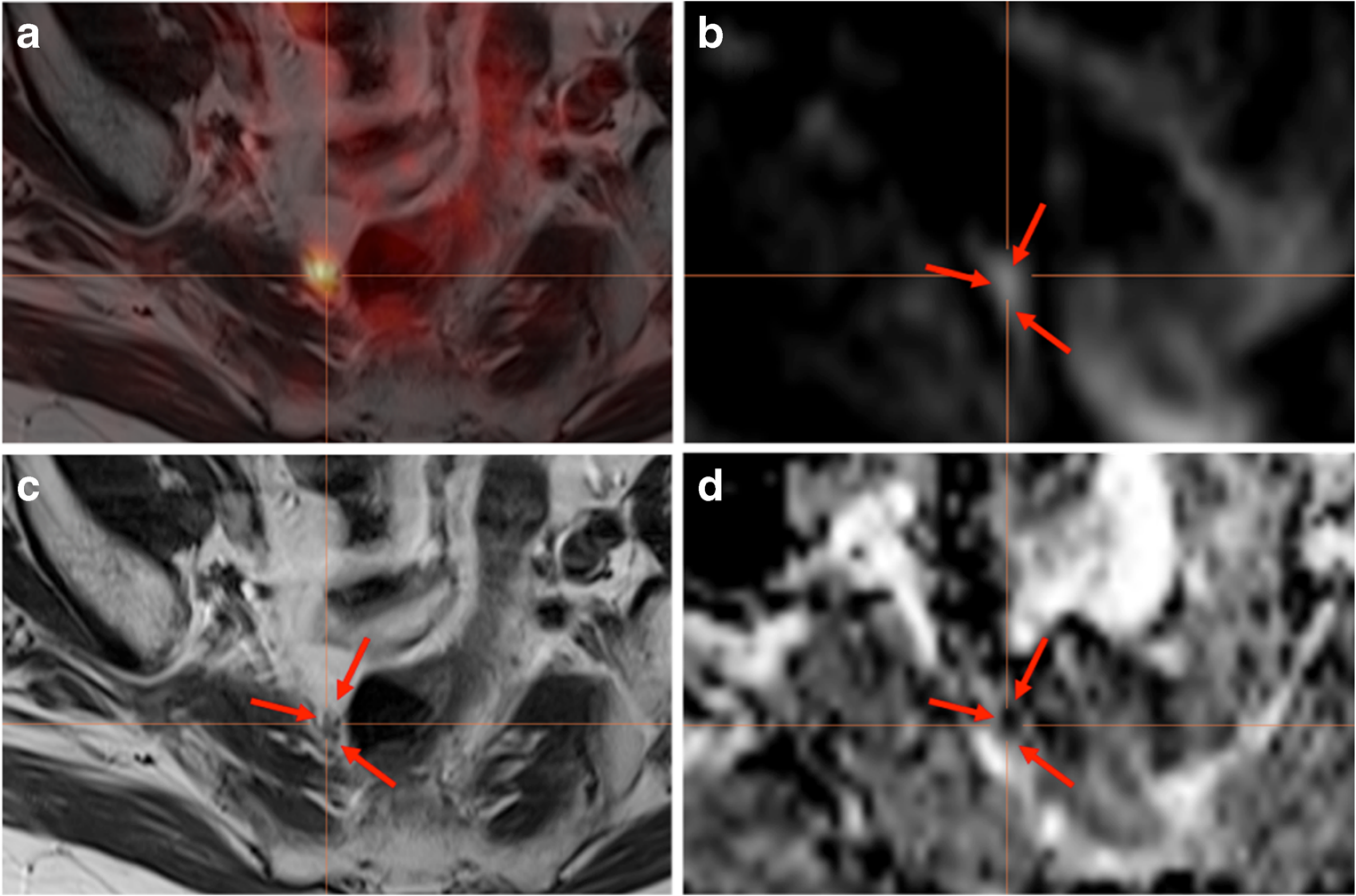
Additional diagnostic value of simultaneous PET/MRI: 77 y/o, post-RPE + RT, pT3a pN1 Gleason 9, PSA at PET/MRI: 1.35 ng/ml: Confirmation of positive [^68^Ga]-PSMA^HBED-CC^ conjugate 11 uptake in a pararectal lymph node (**a**; ca. 5 mm), which is difficult to detect on T2w turbo spin echo sequences (**c**), but has a significant diffusion restriction on DWI b600 images (**b**) and a low apparent diffusion coefficient (**d**), which confirms high cell density in this area. Androgen deprivation therapy was applied. The actual PSA serum level is still <0.2 ng/ml

Radiologic assessment was performed according to RECIST 1.1 criteria. For hybrid assessment, a focal uptake above the surrounding background in a morphologically visible structure (e.g. lymph node any size or bone) or soft tissue in the prostate bed as well as corresponding areas of restricted diffusion capacity in MRI DWI was assessed as a positive finding. Known “false” positive lesions such as slight focal uptake in the area of the paravertebral sympathetic ganglia were ignored [21]. We considered as confirmation of the lesions either a histologic assessment such as biopsy or salvage lymphadenectomy as well as a PSA decline when radiation therapy was directed to the suspicious area. In case of PET/MRI, a positive corresponding diffusion restriction was also taken as a non-invasive confirmation of an area with potential higher cellular density and most likely tumoral tissue. Additional lower values for the apparent diffusion coefficient (ADC) strengthened the confirmation (Fig. 1).

### Tumor board

A standard clinical interdisciplinary tumorboard with the unblinded hybrid imaging results in the mentioned observational period was available for each patient included in this study. Additionally, we retrospectively performed a condensed tumorboard, where only the clinical history, CT/MRI and bone scan results of the respective anonymized patients were presented. The study tumorboard consisted of experts in urology/uro-oncology, pathology and radiation oncology. The real therapy was blinded at the time of discussion. The majority decision of the retrospective tumorboard, blinded to hybrid imaging, was then compared to the real therapies, which were given to the patients according to the institutional tumorboard decision taken out of the clinical records.

### Statistical analysis

Descriptive statistics of categorical variables focused on frequencies and proportions. Means, medians and interquartile ranges (IQR) were reported for continuously coded variables. The Mann-Whitney U test and chi-square test were used to compare the statistical significance of differences in medians and proportions, respectively. Univariable logistic regression was used to assess the predictive ability of PSA for PSMA-PET positivity. Statistical significance was considered at *p* < 0.05. All tests were two-sided. Statistical analyses were performed using STATA v.14.1 (StataCorp LP, College Station, TX, USA).

## Results

### Detection efficacy

Patient characteristics are summarized in Table 1. Overall, 46 (39.3%) patients were evaluated with PSMA-PET/CT and 71 (60.7%) with PSMA-PET/MRI. The median time from RP to BCR was 36 months (IQR 16–72) with 69 (59%) patients having undergone postoperative RT. Median PSA level at the time of imaging was 1.04 ng/ml (IQR 0.58–1.87).

**Table 1:** Clinicopathologic features of 117 patients evaluated with [^68^Ga]Ga-PSMA^HBED-CC^ conjugate 11 ligand PET-CT or PET-MRI for biochemical recurrence after radical prostatectomy for clinically non-metastatic prostate cancer with curative intent

Age (years), median (IQR)	74 (68–76)
Pelvic lymphadenectomy performed at time of RP, n (%)	112 (95.7)
Pathological stage after RP, n (%)	
pT2	46 (39.3)
pT3a	32 (27.4)
pT3b	37 (31.6)
pT4	2 (1.7)
Positive LN at RP, n (%)	11 (9.8)
Positive surgical margins, n (%)	49 (41.9)
Gleason score at RP, n (%)	
6	15 (12.8)
7	56 (47.9)
≥8	46 (39.3)
Post surgery RT, n (%)	69 (59.0)
Adjuvant RT	27 (23.1)
Salvage RT	42 (35.9)
PSMA application (MBq), median (IQR)	180 (167–192)

*IQR* interquartile range, *RP* radical prostatectomy, *LN* lymph node, *RT* radiotherapy, *PSMA* prostate-specific membrane antigen, *MBq* megabecquerel

Overall, 100 of the 117 evaluated patients had at least one PSMA-avid lesion resulting in a detection rate of 85.5%. In 33 (33%) of these patients, both the standard imaging (CT or MRI) and PSMA-PET were positive, while in 67 (67%) patients, the PSMA-PET exclusively provided diagnostic information with no morphologically suspicious correlate. The detection efficacy for PSMA-PET was 65% for a PSA value of 0.2 to <0.5 ng/ml, 85.7% for a PSA value of 0.5 to <1 ng/ml, 85.7% for a PSA value of 1 to <2 ng/ml and 100% for a PSA value ≥2 ng/ml. The median PSA was significantly lower in patients with negative imaging than in patients with positive imaging (0.8 ng/ml, IQR 0.33–1 vs. 1.2 ng/ml, IQR 0.62–2.12; *p* = 0.02).

The different regions of disease recurrence are summarized in Table 2. Overall, 41 (41%) patients had only pelvic or retroperitoneal lymph node metastasis and 22 (22%) had exclusively local recurrence. In 21 (21%) patients, multiple recurrence sites were identified.

**Table 2:** Distribution of [^68^Ga]Ga-PSMA^HBED-CC^ conjugate 11 ligand PET/CT(MRI)-avid lesions in 100 patients with biochemical recurrence after radical prostatectomy

Pelvic lymph nodes only, n (%)	35 (35)
Retroperitoneal lymph nodes only, n (%)	6 (6)
Mediastinal lymph nodes only, n (%)	2 (2)
Bone only, n (%)	12 (12)
Organ metastases only (lungs), n (%)	2 (2)
Local recurrence at site of RP, n (%)	22 (22)
Multiple recurrence sites, n (%)	21 (21)

*RP* radical prostatectomy

### Impact on further treatment decisions

Therapies for patients with positive PSMA-PET/CT/−MRI were as follows: 16 (16%) patients underwent salvage lymphadenectomy, 45 (45%) underwent PSMA-PET-directed RT, 17 (17%) received multiple therapies (either combined chemohormonal therapy and/or androgen synthesis inhibitors) and 16 (16%) received ADT alone. One (1%) patient received chemotherapy without ADT based on the patients’ wishes, and five (5%) patients refused to undergo active therapy despite positive imaging.

**Fig. 2:**
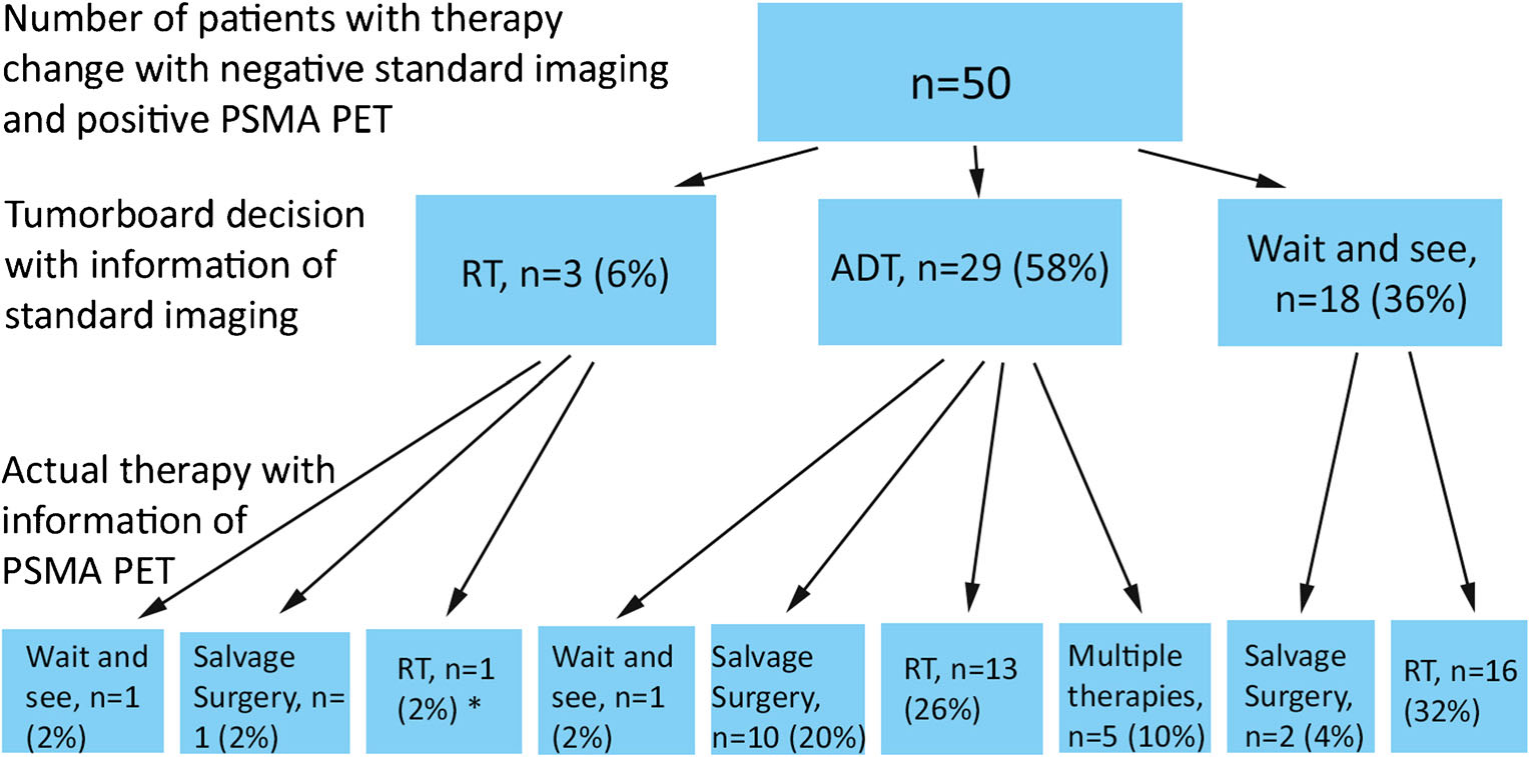
Change of therapy decision in 50 patients with negative standard imaging and positive PSMA-PET. RT = radiotherapy, ADT = androgen deprivation therapy. *1 patient with RT changed to a detected bone metastasis instead of prostate bed

According to our condensed interdisciplinary tumorboard, in 74.6% (50/67) of the patients in whom standard imaging was negative, a positive PSMA-PET changed the therapeutic strategy (*p* < 0.001): 29 (58%) patients with negative standard imaging who would have been referred for ADT, 18 (36%) who would have undergone a wait-and-see strategy, and 3 (6%) who would have undergone a local salvage RT without any evidence of local recurrence (Fig. 2). With the additional information of the PSMA-PET, 86% (43/50) of these patients received a metastasis-directed therapy: 13 patients (26%) received a salvage lymphadenectomy and 30 (60%) received a PSMA-PET-directed RT to pelvic or retroperitoneal lymph nodes or bone metastases. Of the remaining seven (14%) patients, five received a combined chemohormonal therapy due to multiple tumor sites, and in two patients, therapy was changed to a wait-and-see strategy (initially planned as ADT and RT to prostatic bed). For these 50 patients, the median PSA decreased from 0.96 ng/ml (IQR 0.56–1.59) at the time of imaging to 0.17 ng/ml (IQR 0.04–0.41) 3 months after therapy. Six months after therapy, the median PSA for this patient group then slightly increased to 0.23 ng/ml (IQR 0.03–0.73). For a clinical follow-up after 12 months, 41/50 (82%) patients were available until now due to the short follow-up time. The median PSA was calculated as 0.37 ng/ml (IQR 0.04–1.04).

**Fig. 3:**
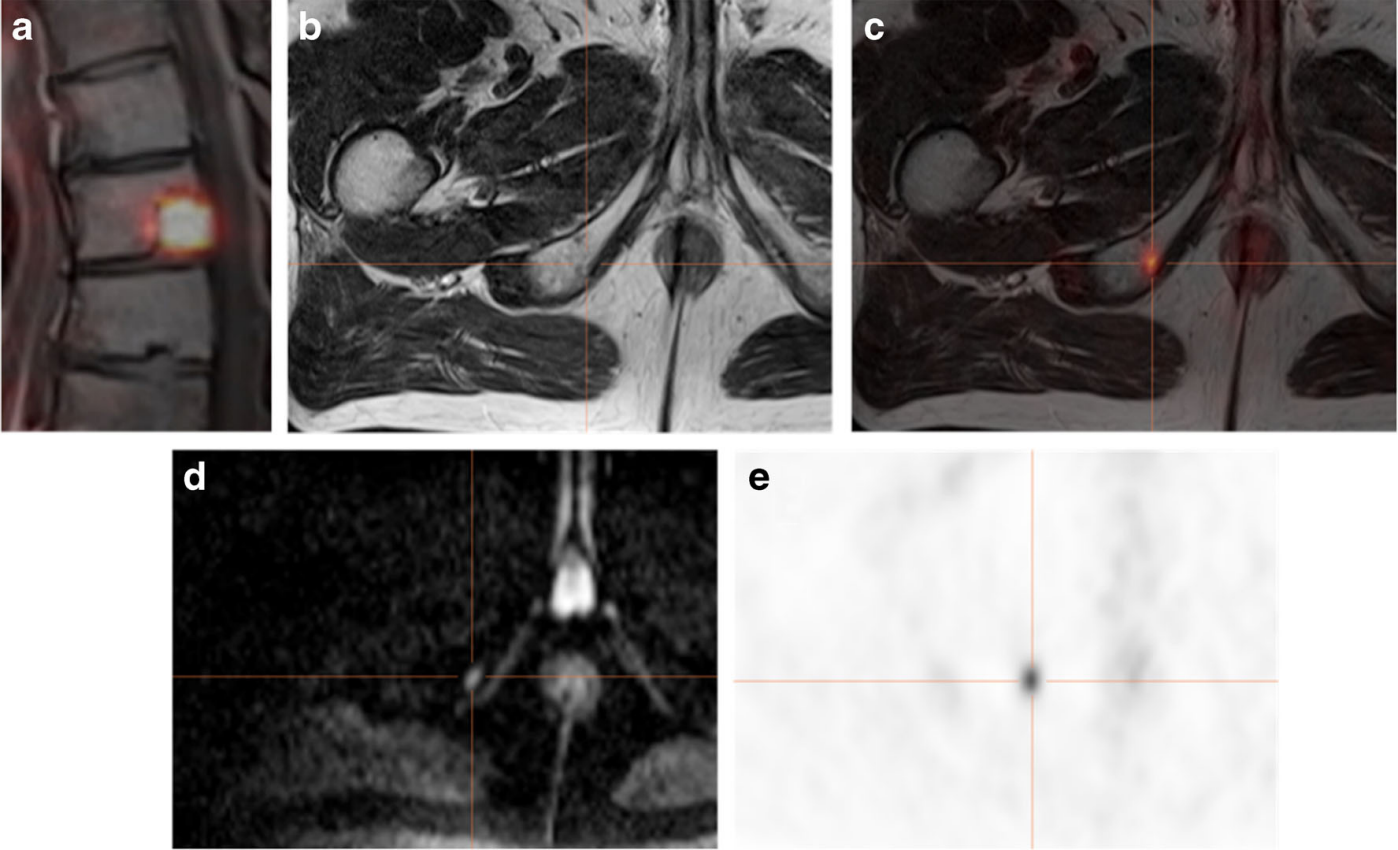
High sensitivity of [^68^Ga]Ga-PSMA^HBED-CC^ conjugate 11 (PSMA)-PET and diagnostic benefit by simultaneous PET/MRI in a 68 y/o patient after radical prostatectomy and radiation therapy of the prostate bed. PSA relapse of 1.7 ng/ml at the time point of PSMA-PET/MRI. Radiation therapy was planned in the thoracic vertebra (**a**; also prospectively positive on MRI) but was then extended to the ischiadic arc. The additional lesion is not seen on T2w sequences (**b**), but the focal PSMA uptake (**c**+**e**) was also confirmed by a focal diffusion restriction on diffusion-weighted b600 sequences (**d**). PSA turned to <0.2 ng/ml after RT

Overall, 39 patients with systemic therapies after positive PSMA-PET were not evaluable for site-specific confirmation of findings; 18 of these patients had simultaneous PET/MRI with a corresponding finding in diffusion-weighted images in all of the PSMA positive lesions (Example Fig. 3). An indirect additional confirmation of the PSMA signal due to a significantly lower ADC demonstrating higher cell density was seen in 66% (*n* = 12) of the respective patients’ lesions (Example Fig. 1).

## Discussion

Treating PC patients with BCR after RP, which is defined as a PSA value of >0.2 ng/ml, is a common but difficult situation. Differentiation of local versus systemic disease and its targeted therapy with the best long-term outcome remains an unmet need. While some patients may benefit from early systemic therapy, others may benefit from local therapy with curative intent. Metastasis-directed therapies are increasingly considered to improve oncologic success while lowering unnecessary side effects. To obtain better outcomes, such therapies need to be administered at lower PSA values while tumor burden is presumably low [5, 8]. Therefore, there is a rising demand for reliable and accurate diagnostic tools. Unfortunately, the detection of metastases at especially low PSA values is not reliable enough with the current guideline-recommended imaging modalities, although choline-based PET [11, 14, 15] has already markedly improved the diagnostic performance of imaging in this setting. In this context, PSMA-PET has emerged as a promising, more accurate method. It has, indeed, been investigated for the staging of recurrent PC after definitive local treatment in retrospective studies [16–18, 22, 23]. However, these studies did not consider all the various issues in the management of these patients. To address this unmet need, we wanted: first to confirm/validate the diagnostic performance of PSMA-PET in patients with BCR after RP; second, to compare the diagnostic performance of PSMA-PET to the current standard imaging in this setting; third, to verify the lesions detected by PSMA-PET as true; and four, to assess how often the PSMA-PET changed the clinical decision-making regarding therapy.

We confirmed the high diagnostic performance of PSMA-PET in patients with BCR after RP. The overall detection rates in retrospective studies published before [17, 18, 23] ranged from 82.2% to 89.5%. Our data are congruent with these mentioned with an overall detection rate of 85.5% at a comparatively lower median PSA level at the time of imaging of 1.04 ng/ml, showing a higher detection efficacy than those published for all known choline-based PET tracers [13, 24, 25]. Our detection rates were from 65% for a PSA value of 0.2 to <0.5 ng/ml up to 100% for PSA values ≥2 ng/ml. As ADT might have an unknown influence on PSMA expression at such early stages of recurrence, we put emphasis on a highly selected and homogenous patient cohort, as we included patients with BCR after RP only, of which a majority (59%) already had postoperative RT. We excluded patients with ongoing ADT as a difference to published data [18]. Nevertheless, it has to be mentioned that an overall detection rate of 85.5% still implies a false negative rate of 14.5%, as it is assumed that any rising PSA level after a nadir of <0.2 ng/ml is representing cancer recurrence.

We found that PSMA-PET detected lesions exclusively with no morphologically suspicious correlate in a majority of the patients. Similarly, Eiber et al. [18] reported that 33% of all the lesions were observed in PSMA-PET only without a suspicious correlate in CT, and that in 25% of the cases, PSMA-PET revealed more lesions than CT. Although this is in line with our data, it seems surprising, as we had significantly more (67%) lesions positive in PSMA-PET only without a suspicious RECIST 1.1 correlate in CT or MRI. Considering the limited role of standard imaging at low PSA levels [11], a reason might be the comparatively lower median PSA in our study, which is nearly half as high as in the study of Eiber et al. This is also true for PSMA-negative findings being assessed as RECIST-positive on CT or MRI that did not occur in our population.

Within our cohort, we found that exact histopathology as a gold standard of proof for PSMA-PET-positive lesions was only available in 16 (16%) patients undergoing salvage lymphadenectomy. However, the observed substantial decrease of PSA in the 45 (45%) patients undergoing PSMA-PET/CT(MRI)-guided RT also provided a regional confirmation. Altogether, 41 of the patients with metastases-directed therapies due to PSMA-PET demonstrated only a slight increase of the median PSA after 6 and 12 month from 0.12 ng/ml to 0.37 ng/ml. This implies a suitable approach for extending therapeutic windows. In 18 (18%) of the remaining 39 patients, a simultaneous PET/MRI was performed demonstrating a correlate with significant diffusion restriction in the PSMA-positive lesion. Although ADC maps are prone to artifacts and patients movement, in 12 (12%) of these patients, a significantly lower ADC signal, showing higher cellularity in the respective area, confirmed the results to a certain extend [26]. Although this is not a histological confirmation, combined PSMA-PET/MRI might have the ability to add diagnostic certainty. In local recurrence, [^11^C]choline PET/MRI demonstrates superior detection rates compared to PET/CT due to the multiparametric MRI protocol [27]. Another study by Freitag et al. using PSMA-PET/CT vs. PSMA-PET/MRI in the evaluation of lymph node metastases demonstrated a significantly higher visibility of PSMA-positive lymph node correlates in MRI-DWI [28]. Taken into account, that studies already reported on the effective use of ADC cut-off values for assessing lymph node malignancy in PC patients [29, 30], it is most likely that PSMA-positive lymph nodes with MRI diffusion restriction have to be considered as malignant, although a dedicated PET/MRI study facing that issue has to be performed separately in a sufficient number of e.g. salvage lymphadenectomy patients. Despite these limitations, a reputable standard of reference was available in 79% (79/100) of PSMA-PET-positive lesions.

We found that PSMA-PET changed the clinical decision-making regarding therapy in 50 patients. In this context, to the best of our knowledge, this is the first study applying a retrospective, interdisciplinary tumorboard, given only conventional imaging results and being blinded to the results of the PSMA-PET and the original therapeutic decision. In this regard, it could be shown, that a positive PSMA-PET had changed the (conventional) treatment management in about three fourths of the patients with negative standard imaging (74.6%), of whom 86% were referred toward metastasis-directed therapies. In other words, 58% (29/50) of the patients with positive PSMA-PET and negative standard imaging did not undergo ADT, delaying its potential risks and side effects, and 36% (18/50) of the patients were not managed with a wait-and-see strategy, which would potentially lead to a progress in the disease and the further growth of metastases, which were not seen in the standard imaging. Instead, 13 of these patients (26%) underwent salvage lymphadenectomy and 30 patients (60%) underwent PSMA-PET/CT(MRI)-guided RT to pelvic or retroperitoneal lymph nodes or bone metastases.

Limitations of this study are its retrospective design which prevents a randomized assessment of the clinical impact of PSMA-PET including clinical follow-up and also provides limited information concerning the details of previous therapies such as the extend of the primary lymph node dissection in this consecutive clinical patient cohort. Due to the applied homogenization of the patient cohort, the final number of patients available for evaluation is reduced. Nevertheless, the results still remain significant. Although the majority of PSMA-positive sites could be acceptably confirmed, a true histopathological gold standard was only available in 16% of the PSMA-positive patients.

## Conclusions

Our data confirm the superior detection rate of disease recurrence sites using PSMA-PET/CT–PET/MRI in patients with BCR after RP for PC compared to the guideline-recommended standard imaging modalities. Additionally, we could demonstrate a clinical impact toward metastases-directed therapies in a cohort of patients with low PSA level recurrences. PSMA-PET may improve early detection, treatment planning and survival in patients with BCR after RP with curative intent and therefore opens windows for targeted therapies before the onset of a systemic approach. Further confirmatory studies with a prospective randomized design are necessary to confirm these preliminary findings.
